# The game theory analysis of homestead use right circulation income distribution under the context of the separation of three powers

**DOI:** 10.1371/journal.pone.0318251

**Published:** 2025-06-03

**Authors:** Junmin Wang, Yajiao Deng

**Affiliations:** School of Business and Administration, Zhongnan University of Economics and Law, Wuhan, China; Indira Gandhi National Tribal University, INDIA

## Abstract

Reasonable distribution of income from the circulation of homestead is the foundation for revitalizing idle rural homesteads. This study constructs a game relationship among villagers, village collectives, and intended parties, with the help of game theory, and discusses the issues about generation logic of circulation income distribution, the bargaining power and so on. It reveals the reasons and conditions for emergence of income distribution, appropriate way of distribution, and the mechanism by which bargaining power affects income distribution. It fills the theoretical gap regarding the generation mechanism and bargaining power of income distribution from homestead land use rights circulation, and lays the groundwork for bridging the ideal and the real about income distribution. The study’s findings indicate that under certain conditions, villagers will choose to share part of the income with the village collective in exchange for the investment in village construction. And compared to income sharing based on circulation price, it based on the area of homestead land is more easily implemented. In addition, the bargaining power of villagers and the village collective determines the result of income distribution, which is closely related to factors such as their negotiation costs and rules. In the process of revitalizing homestead land, it is essential to respect the wishes and rights of villagers, guide cooperation between villagers and village collectives, and reasonably distribute the surplus from cooperation. Additionally, it is necessary to establish reasonable policy rules to prevent distortions in income distribution due to differences in bargaining power.

## Introduction

In the process of urbanization in China, a large number of rural populations have migrated to cities, yet the area of homestead land has been increasing year by year. The long-term decrease in population and increase in homestead has led to the issue of idle homestead. The average idle ratio of rural homesteads is about 20%, representing a huge amount of sleeping assets [1–3]. Idle homestead is not only a waste of land resources but also hinders local socio-economic development. Long-term idle homestead land may also become a collection point for garbage, worsening the ecological environment and living conditions of the village. Moreover, for traditional villages with a long history, the idleness of homestead may lead to the discontinuation of cultural heritage and the destruction of cultural landscapes. Inefficient land use is a common issue both domestically and internationally. By reconstructing the rights of stakeholders, the efficiency of land use can be improved. In Germany, the process is generally led by owners, with government departments playing a service role; Japan also emphasizes public participation, with villagers and guidance representatives forming committees to vote on relevant documents [4]. In China, the implementation of the “three rights separation” policy is a solution proposed in response to the current situation of inefficient homestead land use, which divides the homestead rights into homestead ownership, homestead qualification right, and homestead use right. It breaks through the restrictions of existing laws and regulations on the circulation of homestead use right, and explores the feasibility of the circulation of the homestead use right.

The circulation of homestead use right can increase villagers’ income and strengthen their behavioral capabilities, making them more “freedom”; it can also strengthen village collective economic organizations and improve the welfare level in rural areas; and it can promote rural industrial development, to create more job opportunities [5, 6]. In a word, the circulation of homestead use right is an important approach to achieve integrated urban-rural development and rural revitalization [7–10]. The transformation of value and function is considered the internal reason for promoting the circulation of homestead use rights. As the rural social economy develops, the residential security function of homestead gradually weakens, while its property function gradually becomes more prominent, making the demand for the legal circulation of homestead use rights increasingly urgent [11]. The realization of homestead circulation is influenced and constrained by factors such as land planning, policy framework, property rights structure, and the behavior of relevant entities [3, 12, 13]. Village collectives are considered as the core nodes connecting internal villagers, external capital, and the government, playing roles in information transmission, trust-building, and coordinated management [14]. Villagers are the base point of homestead use rights circulation, and their willingness is affected by factors such as risk perception, regional development levels, wealth disparities, family size, and agricultural dependency [15, 16]. Land is the foundation of farmers’ survival and development. Only by respecting villagers’ needs and intentions and protecting their interests and rights, can villagers be enthusiastic about participating in the transformation. In practice, reform models such as “land ticket” of Chongqin and “exchanging homestead for the house” of Tianjin, have achieved good results. Furthermore, reforms have gradually expanded the transferees and explored various circulation ways including leasing, cooperative management, taking a share, and mortgaging [17]. Driven by strong policy support and huge economic interests, the reform practice of the homestead use rights circulation has achieved certain results [18–20]. At the same time, the issue of the distribution of circulation income has gradually become the focus of social attention [21].

The Guidelines on the Pilot Reform of the Rural Homestead System point out that “to establish and improve relevant mechanisms to ensure that the benefits of homestead expropriation, transfer, withdrawal and economic registration are mainly obtained by farmers”. The circulation of homestead use right is the process in which the villagers give up the use value to realize the exchange value, and the circulation income is the manifestation of its exchange value. Ensuring that the circulation income is primarily obtained by farmers has legal legitimacy. It is generally believed that the village collective, as homestead owner, and the villagers, as the qualification rights holders, are eligible to participate in the distribution of circulation income, but whether the government should directly participate in it is controversial [22, 23]. Due to the lack of uniform and clear rules for the circulation income distribution, the income distribution varies from region to region, which may lead to disputes. Judicial data shows that income distribution has become the core of homestead litigation cases [24]. The rational distribution of income from the circulation of homestead is the basis of realizing the circulation of homestead use right [25]. In the process of homestead use rights circulation, income distribution is predicated on the realization of circulation, which is the result of the behavioral choices of relevant parties, and these choices are driven by interests. A complex mechanism of “income distribution-behavioral choice-circulation result-income distribution” is formed among the relevant parties. Traditional regression methods are only applicable to acyclic directed causal analysis and are not suitable for revealing the complex mechanism behind the income distribution. The “behavior-result-benefit” analysis framework constructed by game theory is conducive to revealing the micro-logic behind the circulation income distribution. Game theory is used to analyze issues about homestead use right circulation involving two or three parties (government, enterprises, village collectives, villagers, or others) in terms of circulation models, subsidy incentives, regulatory constraints, and rights protection [3, 26–28]. Cooperative game theory is considered an effective tool for analyzing income distribution. The income distribution scheme based on the alliance, follows the principle of “distribution according to contribution”, which has a stronger incentive effect, fairness and feasibility [29, 30].

Actual income distribution schemes are often the result of games among the parties, depending on differences in bargaining power rather than value contribution. Distribution schemes based on value contribution are often difficult to achieve spontaneously, creating a gap between the ideal and the real, which is also the reason why disputes easily arise in the income distribution from the homestead use rights circulation in practice. Existing research focuses more on the value evaluation of the income distribution, exploring the basis of fairness and rationality, while neglecting the exploration of generation logic of circulation income distribution. These studies attempt to implement a reasonably fair distribution scheme through an organization (such as local governments) [4, 31]. In essence, it relies on the decisive bargaining power of the organization to achieve a income distribution based on the value contribution of the parties. This kind of assumption creates a principal-agent relationship, leaving room of rent-seeking for the parties. Moreover, when the income distribution depends the organization, the circulation also loses its flexibility and universality. Additionally, to maintain the decisive bargaining power of the organization, it is inevitable to restrict the rights and capabilities of other parties, which may lead to contradictions between the organization and other parties. Policy rule formulation and the allocation of rights are only realistic and feasible when they align with the generation logic of income distribution. Therefore, to achieve a fair distribution of circulation income, it is indispensable to study its generation logic, particularly the bargaining power of the parties.

This paper constructs a game relationship among villagers, village collectives, and intended parties, with the help of game theory, and discusses the issues about generation logic of circulation income distribution, the bargaining power and so on. Firstly, the paper analyzes the roles and relationships of villagers, village collectives, and interested parties in the circulation of homestead use right, and based on reasonable assumptions, establishes the game relationships among them. Then, based on the refinement of the game model, it analyzes the causes and conditions for the generation of income distribution, and examines the way of benefit distribution and the bargaining process. Finally, the study is summarized, and policy recommendations are proposed. It fills the theoretical gap regarding the generation mechanism and bargaining power of income distribution from homestead land use rights circulation, and lays the groundwork for bridging the ideal and the real about income distribution.

## Methodology

### Definition of the concept

#### Circulation of homestead use rights.

The circulation of homestead use rights refers to the situation of the change of homestead use rights. The Guidelines regard “leasing, shareholding, transferring, swapping, and donating” as specific ways of the homesteads use right circulation. Although leasing can make the lessee obtain the right to use the homestead, this right comes from the lease contract, which belongs to the creditor’s right, and does not change the real right (the homestead use rights)Therefore, it is controversial to believe that leasing belongs to homestead use rights - a legal right of property in the way of circulation. In the remaining four ways, all of them have the effect of change of property right. Since transferring is more common than the other three ways, this study establishes a game model based on the transfer process of homestead use right.

#### Circulation income.

In some research, the income from homestead use right circulation is considered to be the change in utility before and after circulation. Since the villagers get the homestead use right for free, the existing research can generally take the income from homestead use right circulation, such as rent and transfer fee and so on, directly as the income from the circulation of homestead. Strictly speaking, the income from the homestead use right circulation is based on the change of homestead use right. No matter the economic income or accounting income, the change of a property right cannot be regarded as zero cost. In addition, the content of the game alliance analysis is also based on the output distribution of each agent’s contribution, rather than the output distribution after deducting the contribution. In addition, the content of the game alliance analysis is also based on the distribution of the output of each subject’s contribution, rather than the distribution of the output after deducting the contribution. Therefore, the income from the circulation of homestead use rights seems to be a more realistic expression. In order to avoid ambiguity, this study continues to use the expression of the previous study - “circulation income” of the homestead use right, which is the consideration obtained in the transfer of the homestead use right.

#### Cooperative surplus.

In this study, cooperation occurs between the villagers and village collectives, and the cooperation surplus is determined by comparing the difference between the maximum and minimum income that farmers may share to village collectives. When the sharing value is minimized, the utility of the village collective is equivalent to that of non-cooperation; When the sharing value is maximum, the utility of farmers is equivalent to that of non-cooperation, and the difference between the two is the surplus of cooperation, which is also the negotiation space between the village collective and farmers.

#### Income distribution and income sharing.

Income distribution is approached from a holistic perspective, defining the share of the total income that different individuals hold. Income sharing, on the other hand, is approached from an individual’s perspective, defining the interest relationships between one individual and others. In the context of the circulation of homestead use rights, income sharing is the manifestation of income distribution at the individual level. Since villagers are the base point of the circulation, the model grants villagers the initiative in income sharing. This approach, which treats income sharing as an option, is more conducive to revealing the generation mechanism of income distribution.

### The game players and their relationship

The process of the homestead use right circulation mainly involves three rights holders. Namely, the village collective as the owner of the homestead, the farmers as the qualification right holder of the homestead, and the interested parties in obtaining the homestead use right. The income from the homestead use right circulation occurs between village collectives and farmers, but the willingness of transfer intention person to pay the consideration for the homestead use right is a prerequisite for the generation of benefits.

#### The villagers.

The villagers are the subject of the homestead qualification right, and homestead use right before transferred. In fact, the villagers occupy and dominate the homestead, and determine whether the homestead use right is transferred and the consideration of the circulation. Farmers have the most complete homestead information, which is conducive to farmers to make reasonable decisions. the villagers have the most complete homestead information, which is conducive to making sound decisions. In the practice of some areas, the decision on whether the homestead use right is transferred and the consideration of the circulation is made by other entities, in place of the villagers, which in fact is a violation of the rights of the villagers, and is likely to cause their resistance. The generation of circulation income is based on the farmers giving up the homestead use value, the circulation income should be obtained by the villagers, and other entities should share it on the basis of not damaging the rights and interests of the villagers and having reasonable reasons. This study argues that the villagers’ status as the subject of rights should be respected, and whether to share income with other subjects belongs to the strategic choice of them, rather than a predetermined result.

#### The village collective.

The village collective is a holistic concept that represents the interests of the villagers as a whole. The village collective is the owner of the homestead, but the village collective cannot directly exercise its rights, and its rights are exercised by the village collective economic organization (or village committee) on its behalf. In fact, the village collective can’t directly occupy and dominate the homestead, but the choice of the village collective has an important impact on the value of the homestead, which is mainly reflected in the village planning, infrastructure construction, village governance and other aspects. The circulation income obtained by the village collective should be prioritized for the construction of the village.

#### Interested parties.

The interested parties, due to the need for residence, is interested in purchasing the homestead use right. Before purchasing, they will compare various schemes that can meet their residential needs. There are many options available to the interested parties, such as buying commercial housing in towns, purchasing the homestead use right in rural areas, renting houses, and long-term renting apartments, etc. Whether they decide to purchase the homestead use right is influenced by its value. In practice, the widespread idleness of rural homesteads is not only due to policy restrictions but also because of the backward rural infrastructure. People with housing needs are more willing to save money to buy houses in towns rather than the homestead use right. Policy restrictions can only be considered as increasing the transaction costs of circulation; if the value of the homestead is large enough, invisible circulation will still occur [32]. Improving the infrastructure of villages can enhance the attractiveness of the right to use homestead land to interested parties. The interested parties obtain the right by paying consideration, which constitutes the circulation income.

#### The relationships among the game players.

Realizing the circulation of the homestead use right can improve the utility of villagers. The consideration obtained in the circulation is the circulation income, and villagers can choose whether to share part of the income to the village collective, which is the strategy of villagers. The village collective can affect the value of the homestead use right. In the study, “whether to carry out road construction” is taken as the representative, which refers to various behaviors of village collectives that can facilitate the circulation of homestead land use rights, and takes it as the strategy of village collectives. The interested parties’ willingness of purchasing the homestead use right is the basis to realize the transfer. In this study, the comparison and strategic space of the interested parties is simplified, using the terms “purchasing the homestead use right in the village” (purchasing in the village) and “purchasing a house in the town” (purchasing in the town) as the strategic choices for the interested parties.

### Hypothesis and parameter settings

**Assumption 1.** The villagers, the village collective, and interested parties are all rational actors, and they are aware of the strategic choices of other subjects and the utility of each outcome.

**Assumption 2.** Before the construction of the road in the village, the consideration that the interested parties are willing to pay for the homestead use right (i.e., the transfer income) is $i_{1}^{\prime }$, and after the road is built, it is *i*_1_. *i*_1_ is related to road construction(r), i.e., $i_{1}=i_{1}^{\prime }+R_{1}(r)$.

**Assumption 3.** When the homestead is transferred, the villagers can choose to share the income with the village collective, and the value of this part of the income being shared is *x*.

**Assumption 4.** The village collective can choose to build the village road, which needs to bear certain risks, as well as a cost in terms of money and time. The value of the money is *r*_2_, i.e., $r_{2}=R_{2}(r)$,, and there is no part of the payoff when the decision is made not to build the road.

**Assumption 5.** The construction of the road will enhance the utility of the homestead use right to the interested parties. The value of the increased utility is *r*_3_, and *r*_3_ is a function of the road construction, i.e., $r_{3}=R_{3}(r)$.

**Assumption 6.** To facilitate the analysis, the study adopts the concept of money metric utility, which uses money to measure the magnitude of utility [33].

### Building a game model

**Fig 1:**
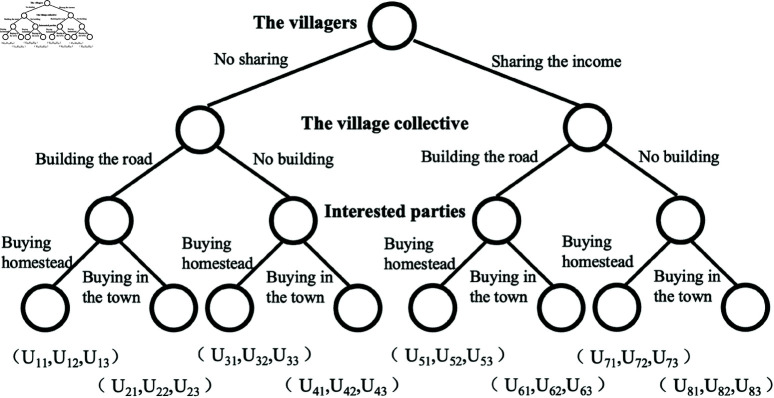
Tripartite game diagram of homestead use right circulation.

In this study, *N* represents the set of game players, and *N* = {the villagers, the village collective, interested parties}. $\theta_{1}$ represents the set of strategies of the villagers, and $\theta_{1}=${no sharing, sharing the income}; $\theta_{2}$ represents the set of strategies of the village collective, and $\theta_{2}=${building the road, no building}; $\theta_{3}$ represents the set of strategies of the interested parties, and $\theta_{3}=${buying the homestead use right, buying a house in the town}. *O* represents the outcome set, that consists of eight outcomes, and *O* = {no sharing, building the road, buying the homestead use right; no sharing, building the road, buying a house in the town; no sharing, no building, buying the homestead use right; no sharing, no building, buying a house in the town; sharing the income, building the road, buying the homestead use right; sharing the income, building the road, buying a house in the town; sharing the income, no building, buying the homestead use right; share the proceeds, no building, buying a house in the town}, using *o* = 1/2/3/4/5/6 /7/8 to refer to these eight outcomes separately. *U*_*on*_ represents the utility of game player *n* (“*n* = 1” represents the villagers, “*n* = 2” represents the village collective, and “*n* = 3” represents the interested person), with the outcome *o*. The game model is constructed as shown in Fig 1.

**Fig 2:**
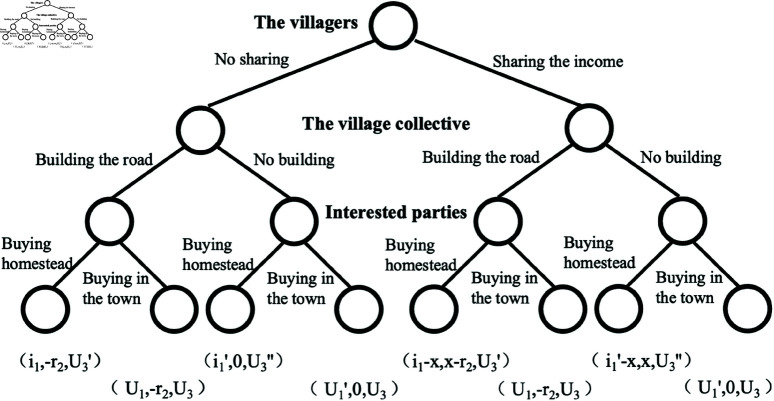
Simplified diagram of the homestead use right transfer.

When the transaction is unsuccessful, the utility of the interested parts is equal, which is denoted by *U*_3_, then, $U_{3}=U_{23}=U_{43}=U_{63}=U_{83}$. When reaching the transaction, the utility of the interested parties is also different, based on whether the road is built or not, so that $U_{3}^{\prime }=U_{13}=U_{53}$, ${U_{3}^{\prime }}^{\prime }=U_{33}=U_{73}$, ${U_{3}^{\prime }}^{\prime }$  +  $r_{3}=U_{3}^{\prime }$ . When the transaction cannot be reached, the utility of the villagers is also affected by road construction, making $U_{1}=U_{21}=U_{61}$, $U_{1}^{\prime }=U_{41}=U_{81}$. When the transaction is reached, both whether the road is built or not and whether the income are shared or not affect the utility of the villagers. *i*_1_ and $i_{1}^{\prime }$ represent the consideration for whether to build the road, and *x* represents the sharing of income. When the village collective economic organization does not receive the shared income, it needs to pay a cost *r*_2_ if the road is built, and its utility is zero if the road is not built. Hereby, the utility set *V* can be simplified by conversion as shown in Fig 2, V={(*i*_1_, −*r*_2_, $U_{3}^{\prime }$), (*U*_1_, −*r*_2_, *U*_3_), ($i_{1}^{\prime }$, 0, ${U_{3}^{\prime }}^{\prime }$), ($U_{1}^{\prime }$, 0, *U*_3_), (*i*_1_−*x*, *x*−*r*_2_, $U_{3}^{\prime }$), (*U*_1_, −*r*_2_, *U*_3_), ($i_{1}^{\prime }-x$, *x*, ${U_{3}^{\prime }}^{\prime }$), ($U_{1}^{\prime }$, 0, *U*_3_)}.

## Results and discussion

### Solving for game equilibrium

#### Game equilibrium without cooperation.

Under non cooperative game conditions, villagers and village collectives cannot reach an enforceable contract. In this scenario, they both have a possession strategy (not sharing profits, not building roads). The game equilibrium depends on the choice of the interested parties. If the utility derived from buying in the village exceeds that from buying in the town, the game equilibrium is (no sharing, no building, buying in the town), and the equilibrium utility is ($i_{1}^{\prime }$, 0, ${U_{3}^{\prime }}^{\prime }$) . Conversely, if the utility of buying in the town surpasses that of buying in the village, the game equilibrium is (no sharing, no building, buying in the town), and the equilibrium utility is ($U_{1}^{\prime }$, 0, *U*_3_). In the case of non-cooperative game, there is no income sharing between villagers and the village collective.

#### Game equilibrium with cooperation.

In the case of cooperative game, game players will collaborate to achieve greater benefits. In this study, since the income distribution occurs between villagers and the village collective, cooperation only takes place between them. Enforceable contracts form the basis of cooperation, and in rural areas, they are typically manifested as institutional norms passed by villager assembly. These contracts constrain the choices of villagers and the village collective, helping them to escape the “prisoner’s dilemma.” The intended parties will always choose the option that maximizes their utility. The choice is related to the utility of purchasing a house in the nearby town (*U*_3_). It is associated with the development level of the nearby town and is independent of the choices made by the game players, and belongs to an external condition. This study firstly refines the game model based on the utility of the intended parties. After eliminating obviously impossible results, the remaining results are compared with the non-cooperative game equilibrium. If there is a result that makes the utilities of both villagers and the village collective better than the non-cooperative game equilibrium, then the villagers and the village collective will cooperate to achieve the result, which is the cooperative game equilibrium. If there is no such result, then the villagers and the village collective cannot reach a cooperation, and the game equilibrium is the same as the non-cooperative game equilibrium.

**Fig 3:**
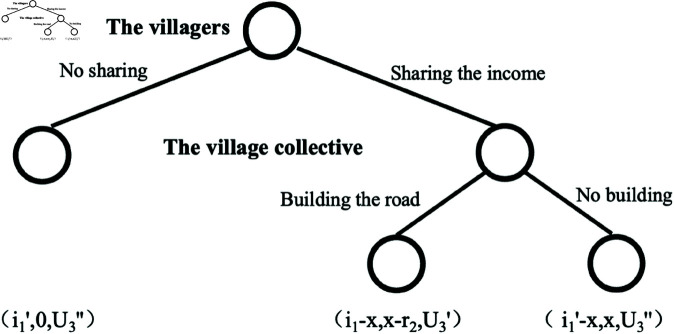
Equilibrium solution graph a.

(1) When $U_{3}<{U_{3}^{\prime }}^{\prime }$, implying $U_{3}^{\prime }>{U_{3}^{\prime }}^{\prime }>U_{3}$, for the interested parties, opting of buying in the village is the dominant strategy. And because the village collective gets the least income from road building when the farmers do not share the income, the rational village collective will avoid this situation, so it will be eliminated and the original game will be refined as shown in Fig 3.

If $i_{1}-x>i_{1}^{\prime }$, and *x*−*r*_2_>0, the village collective economic organizations and the villagers will cooperate to achieve their own maximum benefits. Sign the contract agreement: when the farmers choose to share income, the village collective will choose to build roads. Under these circumstances, the equilibrium choice of the game is (sharing income, building roads, buying in the village), and the equilibrium utility is (*i*_1_−*x*, *x*−*r*_2_, $U_{3}^{\prime }$); When $i_{1}-x\leq i_{1}^{\prime }$, or $x-r_{2}\leq 0$, the villagers and the village collective cannot achieve a Pareto improvement through cooperation, so they cannot reach cooperation. In this case, the game’s equilibrium is (no sharing, no building, buying in the village), and the equilibrium utility is ($i_{1}^{\prime }$, 0, ${U_{3}^{\prime }}^{\prime }$). When $U_{3}<{U_{3}^{\prime }}^{\prime }$, the villagers and the village collective can achieve a Pareto improvement through cooperation. At this time, the value of shared income(*x*) should meet the conditions $i_{1}\;-\;x>i_{1}^{\prime }$, and *x*−*r*_2_>0, namely, $R_{1}(r)>x>R_{2}(r)$. At this point, the income(*x*) shared to the village collective should meet the expenses of the village collective for road construction, and not exceed the land value appreciation due to the infrastructure improvement. Essentially, it can be regarded as that the villagers and village collective share the land value appreciation resulting from the improvement of village infrastructure.

**Fig 4:**
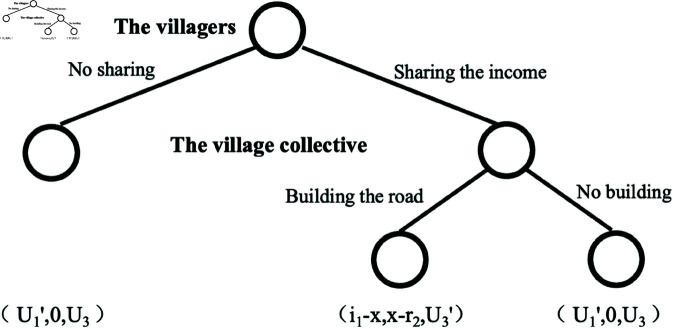
Equilibrium solution graph b.

(2) When $U_{3}^{\prime }>U_{3}>{U_{3}^{\prime }}^{\prime }$, if the village collective economic organization builds the road, the interested parties opt to buy in the village, If the village collective does not build the road, the interested parties will buy in the town. The original game is refined as shown in Fig 4.

If $i_{1}\;-\;x>U_{1}^{\prime }$, and $x\;-\;r_{2}>0$, the village collective and villagers will cooperate to achieve their own maximum benefits. Sign the contract agreement: when the farmers choose to “share income”, the village collective will opt to “build roads”. Under these circumstances, the equilibrium choice of the game is (sharing income, building roads, buying in the village), and the equilibrium utility is ($i_{1}\;-\;x$, $x\;-\;r_{2}$, $U_{3}^{\prime }$); When $i_{1}\;-\;x\leq U_{1}^{\prime }$, or $x\;-\;r_{2}\leq 0$, the villagers and the village collective cannot achieve a Pareto improvement through cooperation, so they cannot reach cooperation. In this case, the game’s equilibrium is (no sharing, no building, buying in the village), and the equilibrium utility is ($U_{1}^{\prime },0,U_{3}$). When $U_{3}^{\prime }>U_{3}>{U_{3}^{\prime }}^{\prime }$, the villagers and the village collective can achieve a Pareto improvement through cooperation. In this scenario, the shared income(*x*) should meet the condition *i*_1_ − $x>U_{1}^{\prime }$, and *x* − *r*_2_>0, namely *i*_1_ − $U_{1}^{\prime }>x>R_{2}(r)$. At this time, the income (*x*) shared to the village collective should meet the expenses of the village collective for road construction, and the benefit retained by the villagers is greater than the benefit of holding the homestead. Its essence can be regarded as that the villagers and village collectives realize the revitalization of idle homestead by improving village infrastructure, and share the benefits of revitalization.

**Fig 5:**
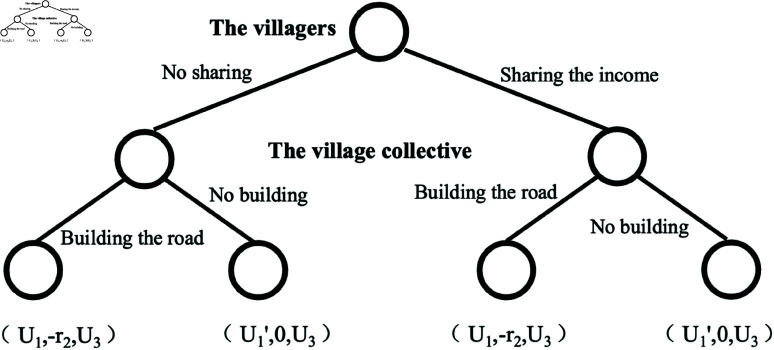
Equilibrium solution graph c.

(3) When $U_{3}\geq U_{3}^{\prime }>{U_{3}^{\prime }}^{\prime }$, there exists a dominant strategy “buying in town” for the interested parties, the original game is refined as shown in Fig 5.

In scenarios, it is impossible to realize transfer, so that there is no income shared. It is not rational for the village collective to build the road because there is no income generated. There is no change in utility of the villagers, no matter what they choose. Here, two game equilibria emerge: (sharing the income/no sharing, no building, buying in the town), with the same equilibrium utility of ($U_{1}^{\prime },0,U_{3}$). Under these conditions, infrastructure construction can’t promote the transaction of homestead and does not generate transfer income. This may be due to the poor local environmental conditions and low develop ability, even if the road construction and other infrastructure construction are upgraded, under a certain time, technical conditions and investment level, it is unable to attract the interested parties to purchase the homestead use right. The migration from peasant settlements can also prove that it is a rational choice for village development to give up areas with poor environmental conditions and lack of development potential and move to settlements.

In summary, under certain external conditions $U_{3}<{U_{3}^{\prime }}^{\prime }$, the villagers can reach cooperation with the village collective, through sharing a portion of the transfer income with the village collective in exchange for improved village infrastructure, thus promoting the circulation (and value-added) of homestead and improving the utility of both parties. Reasonable distribution of income is an internal condition for the establishment of cooperation, and the income shared by the villagers to the village collective should be able to cover the expenditures of the village collective, and the income retained by the villagers should not be less than the utility of non-cooperation.

### The way of sharing income

In the transaction of homestead, if the villagers opt to share part of the income with the village collective economic organization, there are two common ways of sharing. One is based on the proportion of the transaction price(*P*), and the other is based on the fixed amount of the transaction homestead area(*M*). In the game process, the transaction price(*P*) belongs to the private attribute of the villagers and interested parties, and the village collective economic organization does not directly grasp it, while the homestead area(*M*) does not belong to the private attribute.

If the shared income(*x*) is determined based on the transaction price(*P*), the villagers’ report will influent it. Suppose that the actual transaction price of villagers’ circulation is *P*_0_, and the reported price is $P_{0}^{\prime }$, $P_{0}\geq P_{0}^{\prime }$; The income that farmers should share at the actual flow price is *x*_0_, and when the reported price is $P_{0}^{\prime }$, the income that farmers should share is $x_{0}^{\prime }$, *x*_0_ − $x_{0}^{\prime }=\Delta x$. When there is no transfer and villagers choose not to share income, the game equilibrium utility has nothing to do with the sharing income(*x*), and whether the transaction price(*P*) is reported truthfully does not affect the equilibrium utility. When villagers choose to share income and realize circulation, the equilibrium utility is related to the sharing income(*x*), that is, when $U_{3}<U_{3}^{\prime }$, $i_{1}-x>i_{1}^{\prime }$(or $U_{1}^{\prime }$),*x*−*r*_2_>0, whether to truthfully report the circulation price(*P*) will affect the equilibrium utility.

Taking the scenario that $U_{3}<{U_{3}^{\prime }}^{\prime }$, $i_{1}-x>i_{1}^{\prime }$, and *x*−*r*_2_>0, as an example, when the mechanism is implemented truthfully, the game’s equilibrium choice is (sharing income, building roads, buying in the village), and the equilibrium utility is (*i*_1_−*x*, *x*−*r*_2_, $U_{3}^{\prime }$). While the villagers report a trading price of $P_{0}^{\prime }$, the proceeds are shared $x_{0}^{\prime }$. The utility of the outcome (sharing income, building roads, buying in the village) changes to ($i_{1}-x_{0}^{\prime }$, $x_{0}^{\prime }-r_{2}$, $U_{3}^{\prime }$). If $x_{0}^{\prime }-r_{2}=x_{0}-r_{2}-\Delta x>0$, then the game equilibrium is the same (sharing the income, building roads, buying the in the village), and the equilibrium utility changes to ($i_{1}-x+\Delta x$, $x-r_{2}-\Delta x$, $U_{3}^{\prime }$). Since the utility of the villagers is improved in this case, but the utility of the interested parties is not reduced, the villagers has an incentive to lie about the transaction price, and the interested parties does not care about whether the price is reported truthfully. If $x_{0}^{\prime }-r_{2}=x_{0}-r_{2}-\Delta x<0$, then (sharing the income, building roads, buying in the village) is no longer an equilibrium solution to the game, the new game equilibrium is (no sharing the income, no building roads, buying in the village), the equilibrium utility is (*i*_1_, 0, ${U_{3}^{\prime }}^{\prime }$), and the new equilibrium utility is less than the reported equilibrium utility (*i*_1_−*x*, *x*−*r*_2_, $U_{3}^{\prime }$), the utility of each participant is reduced. At this time, if the main body of the villagers is rational and has enough information, the villagers will choose to report truthfully. In the other circulation condition($U_{3}^{\prime }>U_{3}>{U_{3}^{\prime }}^{\prime }$, $i_{1}-x>U_{1}^{\prime }$, *x*−*r*_2_>0), whether villagers truthfully report the circulation price has a similar effect on the game equilibrium utility.

When the villagers share income with the village collective economic organizations, the way of sharing income affects whether the agreement can be truthfully implemented. If the share is made according to the proportion of the transaction price, since the transaction price belongs to the private attribute for the villagers and the interested parties, farmers have an incentive to misrepresent the transaction price, while the interested parties do not have an incentive to reveal it, which induces the generation of intentionally underreported prices. However, if the revenue sharing is carried out according to the homestead area(*M*), the game mechanism is more likely to be implemented truthfully, because the homestead area(*M*) is not a private attribute.

### Bargain between villagers and the village collective

In order to realize the transfer of idle homestead, the villagers and the village collective reach an agreement to promote the construction of infrastructure in the village through sharing part of income(*x*). This study analyzes the bargaining process between the villagers and the village collective in the case of ($U_{3}^{\prime }>U_{3}>{U_{3}^{\prime }}^{\prime }$, $i_{1}-x>U_{1}^{\prime }$, *x*−*r*_2_>0) as an example. At this time, the equilibrium choice of the game is (sharing the income, building roads, buying in the village), and the equilibrium utility is (*i*_1_−*x*, *x*−*r*_2_, $U_{3}^{\prime }$). The algebraic transformation of the equilibrium condition yields x∈(*r*_2_, *i*_1_ − $U_{1}^{\prime }$). The competition of the shared income(*x*) can be analyzed by a two-person bargaining model, where (*r*_2_, $i_{1}-U_{1}^{\prime }$) is the bargaining range of *x* [34]. If *a* is a cooperative residue, then $a=i_{1}-U_{1}^{\prime }-r_{2}=i_{1}^{\prime }-U_{1}^{\prime }-R_{2}(r)+R_{1}(r)$. $i_{1}^{\prime }-U_{1}^{\prime }$reflects the transfer potential for homestead before the road improvement, and $-R_{2}(r)+R_{1}(r)$ reflects the cost and benefit of constructing the road. The essence of the bargaining between the villagers and village collectives for the shared income(*x*) is the competition for the cooperation surplus(*a*).

**Fig 6:**
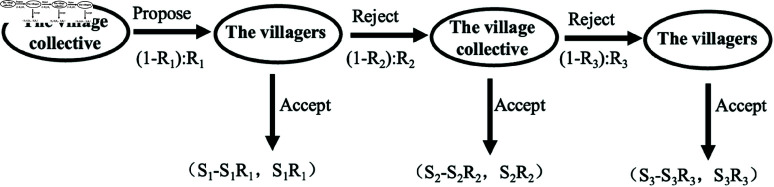
Three-stages bargaining analysis between villagers and the village collective.

The negotiation process is simplified into a three-stage bargaining procedure as shown in Fig 6. The village collective initially proposes a distribution scheme for the cooperative surplus(*a*). If the villagers agree to this scheme, it is implemented; if there is dissent, the villagers’ assembly is convened to propose a new distribution scheme. For the distribution plan proposed by the farmers, if the village collective agrees with the villagers’ proposal, it will be implemented; if not, the village collective will put forward the final distribution scheme, which the farmers can choose to adopt and implement, or not adopt and the cooperation breaks down, and the next round of negotiations will not be conducted. Losses occur in the process of rejecting the other party’s allocation plan and proposing a new one. *S*_*t*_ is defined as the actual distributable cooperative surplus after deducting the loss in the t-round distribution scheme, then $S_{t}=S_{1}-n(\delta_{1}+\delta_{2})$, *t* = 2*n* + 1; or $S_{t}=S_{1}-n(\delta_{1}+\delta_{2})+\delta_{2}$, *t* = 2*n*. The upper limit of *t* is $S_{t}\geq 0$ and *S*_*t* + 1_<0. $\delta_{1}$ and $\delta_{2}$ respectively represent the losses generated when villagers and villages collectively reject each other’s proposals and propose their own plans. In the distribution plan proposed by the village collectives in the first round, the distribution ratio between the village collectives and villagers is $(1-R_{1}):R_{1}$. At this time, the cooperative surplus does not lose, and the distributable surplus is *S*_1_ = *a*. In the second round, in the distribution plan proposed by the farmers, the distribution ratio between the village collective and the villagers is $(1-R_{2}):R_{2}$. At this time, the distributable cooperative surplus is lost due to negotiation, $S_{2}=S_{1}-\delta_{1}$. In the third round, the distribution ratio of the distribution plan proposed by the village collective is $(1-R_{3}):R_{3}$, and the total distributable surplus is $S_{3}=S_{2}-\delta_{2}$.

If the game players have complete information, a reasonable distribution would be adopted in the first round, so as to avoid unnecessary losses in the subsequent rounds. Using the inverse method, the remaining distribution scheme of the third round is ($S_{3}-S_{3}R_{3}$, $S_{3}R_{3}$). Then in the second round, if the income accepted by the village collective are greater than those not accepted, the village collective will choose to accept the scheme of the villagers, that is, $S_{2}-S_{2}R_{2}\geq S_{3}-S_{3}R_{3}$ and $S_{3}R_{3}+\delta_{2}\geq$ $S_{2}R_{2}\geq S_{3}R_{3}$. In the first round, if the income accepted by the villagers are greater than the income rejected, the villagers will choose to accept, at which time $S_{1}R_{1}\geq S_{2}R_{2}$, and the surplus obtained by the village collective satisfies $S_{2}-S_{2}R_{2}+\delta_{1}\geq S_{1}-S_{1}R_{1}\geq S_{2}-S_{2}R_{2}$. If the proposer only considers his own interests and whether the proposal can be passed when proposing the proposal, the proposer will attribute all the savings to himself. Therefore, in the first round of the game, in order to reduce subsequent losses, the village collective proposed a reasonable distribution plan as ($a-\delta_{2}$, $\delta_{2}$), the rational choice of farmers is to accept, and the game reaches equilibrium.

**Fig 7:**
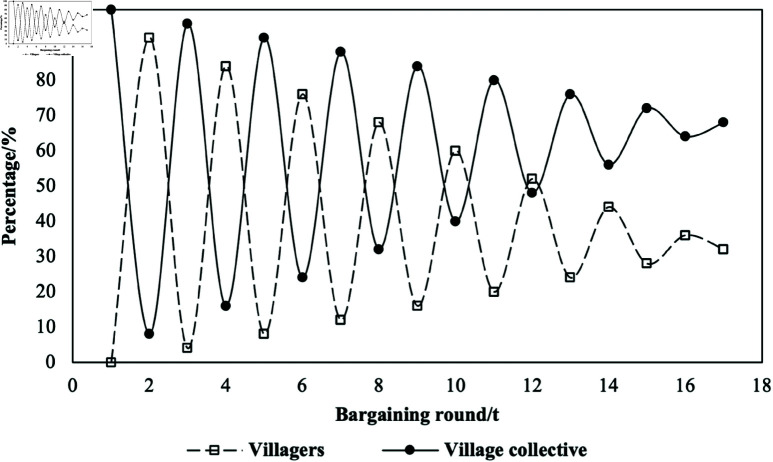
Equilibrium utility graph of the players at different bargaining round.

If the game does not end in the third stage but extends to five, seven, even 2*n* + 1 stages, the game equilibrium is ($S_{2n+1}+n*\delta_{1}$, $n*\delta_{2}$), by mathematical derivation. If the game has four, six or 2*n* rounds, and the farmer proposes the final plan, then *R*_2*n*_ = 1, it can be seen by mathematical deduction that the equilibrium of the game is still reached in the first stage and the equilibrium plan is ($S_{1}-S_{1}R_{1}$, $S_{1}R_{1}$)=($n*\delta_{1}$, $n*\delta_{2}-\delta_{2}+S_{2n}$). Additionally, the residual allocation scheme may also be proposed by the farmer first, at which point the analysis process and results are similar and will not be repeated. In the bargaining game model, the main factors influencing the game equilibrium are the number of stages *t* = 2*n*(or 2*n* + 1), and the losses $\delta_{1}$ and $\delta_{2}$ that farmers and villages collectively reject and propose new solutions. The relationship between the shares of the villagers and the village collective and the number of game stages(*t*) is as shown in Fig 7 (assigning values of 100, 8, and 4 to *S*_1_, $\delta_{1}$ and $\delta_{2}$, respectively). The scatter “Villagers” (“Village Collective”) indicates the share of the villagers (village collective) as the game stage(*t*) increases when the village collective acts first. The total number of stages of the game is determined by $S_{t}\geq 0$ , *S*_*t* + 1_<0, and the total stage number is 17 when the village collective moves first.

The influence of the number of stages(*t*) on the equilibrium scheme is reflected in two aspects: the odd even number and the size of *t*. When *t* is odd, the proposal in the round of *t* is made by the village collective, and all distributable surplus in the round of *t* will belong to the village collective, i.e., *R*_*t*_ = 0. Conversely, when *t* is even, the proposal in the round of *t* is made by villagers, and all distributable surplus will belong to the villagers, i.e., *R*_*t*_ = 1.

The effect of the parity of the game stage(*t*) on the equilibrium share is shown in the figure as the fluctuations of the scatter point. The influence of the number of game stages(*t*) on the equilibrium scheme is also reflected in the stage value. The larger the number of game stages, the smaller *S*_*t*_ becomes, and the smaller the impact of who proposes the program in the t-round is. The impact of the value of *R*_*t*_ (0 or 1) becomes lower, and the greater the impact of the respective losses $\delta_{1}$ and $\delta_{2}$ of the villagers and the village collectives on the equilibrium allocation program. It can be seen from the figure that with the increase of *t*, the absolute value of the fluctuation of the scatter decreases. The equilibrium solution is also related to the relative magnitude of the losses $\delta_{1}$ and $\delta_{2}$ of the villagers and village collectives. When the value of *t* is determined, the higher the losses of the participants in rejecting and proposing a new scheme, the lower their share in the equilibrium scheme. Moreover, with the increase of *t* value, *S*_*t*_ gradually becomes smaller, and the impact of the attribution of *S*_*t*_ on the equilibrium of the game gradually decreases, and the respective equilibrium shares of the farmers and the village collectives gradually converge to the inverse ratio of their respective losses.

Policies and rules can affect the bargaining power of villagers and the village collective, mainly reflected in the negotiation rules and the top-level design of circulation. The negotiation rules between the village collective and villagers, especially the way of negotiation, will affect their bargaining power. The village collective can choose to negotiate with individual villager or with villager group. When the village collective chooses to negotiate with the villager group, the loss of the villager group reaching an agreement to reject the village collectives’ offer and propose a new offer is much greater than the loss of the village collective to reject the villager group’s offer and propose a new offer. It implies that between the villager group and the village collective, the distribution of cooperative surplus will tend to the village collective rather than the villager group, when the village collective only considers its own interests and whether the cooperation can be reached. Compared to the villager group, individual villagers have much less loss in rejecting the offer and proposing a new, and thus have stronger bargaining power when negotiating with the village collective. If the village collective chooses to negotiate with individual villagers, it will be at a disadvantage, and the cooperative surplus will be heavily skewed towards the individual villagers. Moreover, individual villagers have their own differences. There is significant psychological or social pressure for some villagers to reject the village collective’s proposal, but it may be much smaller for others. The pressure can be considered a form of loss. Due to the differences of individual villagers, when the village collective chooses to negotiate with individual villagers, the negotiation result is often affected by their individual attributes, resulting in differentiated and unfair distribution schemes. The difference is often manifested as those villagers who cooperate more with the village collective suffer more, while those who dare to oppose gain more advantages.It will further induce individual villagers to oppose the village collective’s plans, making the situation faced by the village collective increasingly difficult. To avoid such situations, the village collective should preferably choose to reach executable agreements with the villager group, rather than negotiating with individual villagers.

Additionally, the convening process and location of the villagers’ assembly can impact bargaining power. The more remote the location of the villagers’ assembly and the more cumbersome the process, the higher the negotiation costs for the villagers, and the lower their bargaining power. At the villagers’ assembly, the more thorough the communication among villagers and the more accurate the summary, the higher the efficiency of reaching consensus, the lower the negotiation costs for the villager group, and the stronger the bargaining power. The impact of the top-level design of circulation on bargaining power is mainly reflected in aspects such as the method of circulation and the dominance. The bargaining power of villagers under unified circulation and decentralized circulation is different. In the case of unified circulation, the negotiation costs for villagers to reach consensus is greater, and their bargaining power is lower, compared to decentralized circulation. The dominance of the circulation will affect the bargaining power. In a circulation model dominated by villagers, the village collective has a weaker influence on the circulation, so its bargaining power is also weaker, compared to the model dominated by village collective. The supervision of the village collective by villagers also affects the distribution of profits. The village collective is supervised by the villagers, so members of the village collective may, when weighing interests, concede some of the benefits that the village collective could obtain to some villagers in order to secure their support for their own re-election. It essentially represents a betrayal of the interests of the village collective by its members and does not change the difference in bargaining power between the village collective and the villagers.

### Analysis of reform policies on income distribution

The income distribution system of homestead land use rights circulation is explored in pilot areas. In Yicheng City, all circulation incomes are attributed to the villagers, with the village collective not participating in the income distribution. Rational ways for the village collective to participate in income distribution are explored in more areas, where it is clearly required that the village collective should use the income obtained for village construction, rural industrial development, and other related areas. The management fee for homestead use right circulation is a common way for the village collective to participate in income distribution. The management fee is charged based on the circulation price in Wenchang City, Deqing County, and other regions, which is based on the area and location of the homestead in Tongyuan Town. In areas such as Qiongshan District of Haikou City and Jiangshan City, there are no specific regulations on the amount of management fee; instead, it is determined through negotiations between the village collective and the villagers, guided by the local government. In addition, the standardization of village collective revenue management has also attracted attention. Ensuring villagers’ rights to be informed, to participate, and to supervise helps to regulate the management of village collective.

Differences in understanding the generation logic of income distribution are the reasons for the differences in policies and systems across various regions. Allocating all circulation income to villagers can effectively protect their interests, but it also suppresses the village collective’s willingness to take action to promote the circulation and value-added of homestead. The village collective can participate in income distribution by cooperating with villagers, which not only does not harm the interests of the villagers but can also improve their utility. Policies that limit the application of village collective income can be seen as an offer made by the village collective. At this point, as long as villagers choose to share income, cooperation can be achieved. When the village collective charges the management fees, it is easier to implement truthfully based on area and location, rather than circulation price. Unifying the income distribution by the government can reduce distortions in distribution caused by differences in bargaining power, but it also suppresses the potential for circulation. Determining income distribution through negotiations between villagers and the village collective can better realize the potential of circulation. However, differences in bargaining power between villagers and the village collective may lead to distortions in income distribution. A compromise approach is to determine a reasonable range for distribution, by government, and each village, based on actual conditions, determines the final income distribution plan through negotiations between the village collective and the villagers. Negotiation rules have a significant impact on the bargaining power of the village collective and villagers. Standardizing the negotiation process and the behavior of the village collective is conducive to improving negotiation efficiency and protecting the rights and interests of villagers.

## Conclusions and policy recommendations

### Conclusions

This study constructs a game relationship among villagers, village collectives, and intended parties, with the help of game theory, and discusses the issues about generation logic of circulation income distribution, the bargaining power and so on. It reveals the reasons and conditions for emergence of income distribution, appropriate way of distribution, and the mechanism by which bargaining power affects income distribution. The study reveals that:

(1) Cooperation is the prerequisite for the generation of income distribution. The cooperation between villagers and the village collective is affected by the external environment. The external environment influences the choices of the intended parties, which in turn affects the occurrence of cooperation and circulation income. Under certain conditions $U_{3}^{\prime }>U_{3}$, the villagers can reach cooperation with the village collective and agree to share a portion of the transfer income to the village collective, in exchange for the village collective to carry out infrastructure development, so as to promote homestead use right transfer (or increase the circulation income). The shared income(*x*) with the village collective should cover its expenditure costs, and the income retained by villagers should not be lower than the utility without cooperation.
(2) The income distribution way based on the area of homestead is easier to implement truthfully compared to the way based on the circulation price. It is because the circulation price is private information between the villagers and the intended parties. The income distribution way based on the price may lead to villagers deliberately underreporting the price, in contrast the way based on the area of homestead does not.
(3) The final income distribution is related to the bargaining power of the villagers and the village collective, as well as the rules of negotiation. Regardless of the number of expected negotiation stages, they will reach an agreement in the first stage of the negotiation. And the result of the negotiation is related to the expected number of negotiation stages, their respective bargaining losses, and the ownership of the right to propose the final plan. The fewer the expected negotiation stages, the greater impact of the final proposal right on the bargaining results are inversely proportional to the approaches to their respective losses.

### Policy recommendations

To promote the circulation of homestead use rights and achieve a rational distribution of circulation income, this study puts forward the following recommendations:

(1) Efforts should be encouraged and guided to establish cooperation between the village collective and the villagers. The village collective’s contribution to the circulation (and value-added) of homestead is the basis for the participation in the income distribution. Clarifying the use of the income obtained by the village collective can facilitate cooperation. The village collective should obtain income based on area and location rather than circulation prices.
(2) Under different external conditions, the most suitable income distribution ratios vary. The more explicit the income distribution ratios stipulated by the government, the more they can reduce the distortion in income distribution caused by bargaining power, but the more circulation possibilities suppressed. Ambiguous income distribution ratios are conducive to reaching cooperation, but they are prone to causing distortions in income distribution. A compromise solution is for the government to determine a reasonable range of income distribution, and the specific ratio is then negotiated by the villagers and the village collective.
(3) The bargaining power between villagers and the village collective should be balanced through the optimization of rule to reduce distortions in income distribution. Negotiation rules have a significant impact on the bargaining power of both the village collective and the villagers. By standardizing the negotiation process and regulating the behavior of the village collective in negotiations, the efficiency of negotiations can be improved and the rights and interests of the villagers can be protected.
